# The ensured proliferative capacity of myoblast in serum-reduced conditions with Methyl-β-cyclodextrin

**DOI:** 10.3389/fcell.2023.1193634

**Published:** 2023-05-12

**Authors:** Tomoka Katayama, Yuta Chigi, Daiji Okamura

**Affiliations:** ^1^ Department of Advanced Bioscience, Faculty of Agriculture, Kindai University, Nara, Japan; ^2^ Institute of Advanced Medical Sciences, Tokushima University, Tokushima, Japan

**Keywords:** myoblast, myotube, Methyl-β-cyclodextrin, cholesterol, apoptotic cell death, proliferation, differentiation

## Abstract

To produce muscle fibers for cultured meat on a large scale, it is important to expand myoblasts in a serum-reduced or serum-free medium to avoid cost, ethical, and environmental issues. Myoblasts such as C2C12 cells differentiate quickly into myotubes and lose their ability to proliferate when the serum-rich medium is replaced with a serum-reduced medium. This study demonstrates that Methyl-β-cyclodextrin (MβCD), a starch-derived agent that depletes cholesterol, can inhibit further differentiation of myoblasts at the MyoD-positive stage by reducing plasma membrane cholesterol on C2C12 cells and primary cultured chick muscle cells. Furthermore, MβCD efficiently blocks cholesterol-dependent apoptotic cell death of myoblasts, which is one of the mechanisms by which it inhibits the differentiation of C2C12 myoblast cells, as dead cells of myoblast are necessary for the fusion of adjacent myoblasts during the differentiation process into myotubes. Importantly, MβCD maintains the proliferative capacity of myoblasts only under differentiation conditions with a serum-reduced medium, suggesting that its mitogenic effect is due to its inhibitory effect on myoblast differentiation into myotube. In conclusion, this study provides significant insights into ensuring the proliferative capacity of myoblasts in a future serum-free condition for cultured meat production.

## Introduction

The current meat system relies on industrial livestock farming, which faces several challenges in achieving the Sustainable Development Goals (SDGs). These challenges include excessive consumption of water and grain, deforestation to secure breeding space, and water pollution from livestock excreta (Datar and Betti, 2010) (Post et al., 2020). In addition, the release of large amounts of methane gas through the methane fermentation of livestock excreta and cattle burps has a negative impact on global warming (Humpenöder et al., 2022). To address these challenges, there is an urgent need to establish a sustainable meat production system, particularly given the expected population growth. “Green meat” made from plant proteins and cultured meat made by culturing and organizing muscle cells from livestock are two potential solutions. However, despite the potential of cultured meat to overcome the problems faced by industrial livestock farming, it still faces several challenges in terms of social implementation.

To efficiently expand animal cells in cultures, such as muscle cells and cancer cells, it is still necessary to supplement the culture medium with fetal bovine serum or recombinant proteins, which can be costly (Brunner et al., 2010) (Wong et al., 2020) (Jang et al., 2022). While the main goal of cultured meat is to reduce reliance on and mitigate environmental damage caused by livestock, the use of fetal bovine serum in its production contradicts this objective. While serum-free media that incorporate growth factors like FGF-2, EGF, or IGF-1 have been developed to promote muscle cell proliferation, the production of cultured meat on a large scale necessitates a culture technology, that is, not only serum-free or serum-reduced but also independent of growth factors, which are the primary drivers of cost. To date, no report has been discovered of a technology that has achieved this objective (Jang et al., 2022) (Lawson and Purslow, 2000) (Molnar et al., 2007).

**FIGURE 1:**
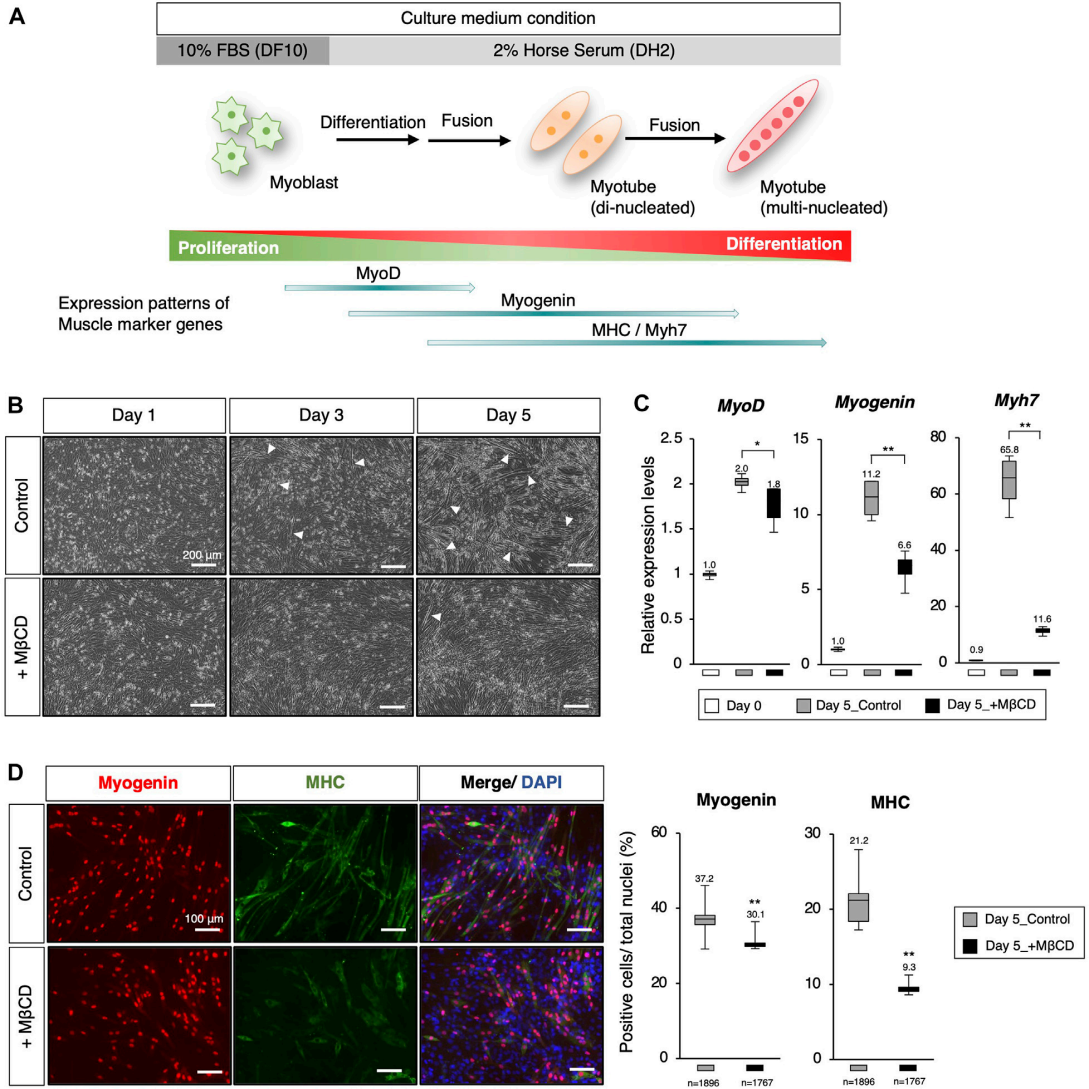
Inhibition of myoblast differentiation with Methyl-β-cyclodextrin (MβCD) in C2C12 cells. **(A)** Schematic representation of myogenic differentiation in C2C12 myoblast cells under serum conditions and the expression patterns of muscle markers during this process are illustrated. The growth of myoblast cells in culture requires fetal bovine serum (FBS) under serum-rich conditions (10% FBS/DMEM as DF10). Under serum-reduced conditions (2% Horse Serum/DMEM as DH2), myoblasts quickly differentiate into myotubes and lose their ability to proliferate. This process involves the first fusion between myoblasts to form di-nucleated myotubes, followed by continuous fusion to form multi-nucleated myotubes. **(B)** Representative images of cultured C2C12 cells undergoing differentiation under DH2 culture conditions with and without MβCD (3 mM) are presented. Elongated cells, which are characteristic of differentiating myotubes, are indicated by white arrowheads. The scale bar is 200 μm. **(C)** Quantitative PCR analysis to evaluate the expression of muscle markers in growing C2C12 cells cultured in DF10 (Day 0), differentiating C2C12 cells cultured in DH2 (Day5_control), and DH2 with MβCD (Day5_+ MβCD). The error bars represent the standard deviation (n = 4, biological replicates). **(D)** Immunofluorescence for Myogenin and MHC (Myosin Heavy Chain) protein expression, which confirms myotube differentiation in C2C12 cells on day 5 of induction with DH2 medium. Nuclei were counterstained with DAPI. The scale bar is 100 μm. The frequency of Myogenin- and MHC-positive cells was calculated as a percentage of total nuclei. The total number of counted cells is indicated in the graph. Error bars indicate s.d. and one-way ANOVA followed by the t-test with **p < 0.01, *p < 0.05.

Skeletal muscle fibers organize in bundles, forming muscle tissue, which is the main component of meat. The source cells for muscle fibers are highly proliferative myoblasts, which are the only proliferative cells in embryonic muscle differentiation after their fate has been determined as muscle (Wong et al., 2020). Mononuclear myoblasts fuse with adjacent myoblasts to form myotubes (Karalaki et al., 2009). During this process, some myoblasts undergo apoptotic cell death, and the membrane components of these dead cells can induce neighboring myoblasts to undergo cell fusion (Lehka and Rędowicz, 2020). Subsequent fusion of myotubes or mononuclear myoblasts to myotubes leads to differentiation and maturation into multinucleated myotubes (Lehka and Rędowicz, 2020). C2C12 cells, which are mouse skeletal myoblasts commonly used as a model for skeletal muscle differentiation, continue to proliferate as myoblasts in a serum-rich medium containing 10% fetal bovine serum (DF10) (Wong et al., 2020) (Lawson and Purslow, 2000) (Yaffe and Saxel, 1977a). However, switching to a serum-reduced medium containing 2% horse serum (DH2) triggers differentiation, followed rapidly by differentiation into myotubes (Wong et al., 2020), (Lawson and Purslow, 2000), (Burattini et al., 2004) (Figure 1A). To produce large quantities of muscle fibers for cultured meat, it is important to expand myoblasts in a serum-reduced or serum-free medium with the differentiation inhibitor to avoid cost, ethical, and environmental issues (Wong et al., 2020), (Jang et al., 2022).

In this study, Methyl-β-cyclodextrin (MβCD), a cyclic oligosaccharide derived from starch, was identified as a potent inhibitory agent on the differentiation of C2C12 myoblast cells in serum-reduced medium. Furthermore, MβCD was shown to significantly maintain the proliferative capacity of myoblasts under serum-reduced medium conditions. Mechanistically, it was found that apoptosis of C2C12 cells during differentiation occurs in a cholesterol-dependent manner, and the addition of MβCD for cholesterol depletion strongly suppresses the cell death of these cells. The reduction of cell death by MβCD may be one of the molecular mechanisms for its inhibitory effect on myoblast differentiation into myotubes. The effects of MβCD on the inhibition of myoblast differentiation and proliferative capacity were also confirmed in primary cultures of chick embryo skeletal muscle. These findings provide important clues for developing technology for efficient myoblast expansion under future serum-free media conditions.

## Materials and methods

### Culture of C2C12 cells for proliferation and differentiation

C2C12 cells (Yaffe and SAXEL, 1977b), an immortalized mouse myoblast cell line (ECACC: 91,031,101), were cultured for rapid proliferation in a “DF10” medium composed of 10% Fetal Bovine Serum (Gibco, 10270-106) and DMEM medium (nacalai tesque, 08458-45) supplemented with Penicillin-Streptomycin Mixed Solution (P/S) (1x) (nacalai tesque, 26253-84). The cells were passaged every 3-4 days with TrypLE (Gibco, 12604-013) at a split ratio of 1:5. C2C12 cells are capable of differentiating into myotubes under low serum conditions (Burattini et al., 2004), (Andrés and Walsh, 1996). For induction of differentiation, 3.0 × 10^4^ cells were seeded onto one well of a 4-well plate (Nunc) in the DF10 medium. At 2-3 days after seeding, when the cell confluency reached 90%–100%, the medium was changed to a “DH2” medium, which is a reduced-serum condition composed of 2% Horse Serum (H1138, Sigma)-contained DMEM supplemented with P/S (1x) with Methyl-β-cyclodextrin (MβCD) (Sigma-Aldrich, 332615) at a concentration of 3 mM. The solvent, sterile water, was added for the control. In the case of cultivation for 5 days, the DH2 medium was changed on day 3.

### Primary culture of embryonic chick myogenic cells

Thigh muscle tissue was dissected from 10-day-old chick embryos, typically obtained from a certified poultry farm, using fine forceps. The femurs were surgically removed from the thigh muscle tissue, and the isolated thigh muscle tissue was minced with fine forceps and washed in PBS twice. The minced tissue was then digested with 2 mg/ml collagenase Type II (Worthington Biochemical Corporation, WOR-CLS2) in HBSS (nacalai tesque, 09735-75) at 37°C for 40-60 min, depending on the detachment of cells, with pipetting every 10–15 min. The dissociated myogenic cells were passed through a 40 μm cell strainer (Corning, 431750) for single-cell suspension, and 5.0 × 10^5^ dissociated cells were plated onto one well of a 4-well plate precoated with 0.2% gelatin in DF10 medium for stable attachment. On the following day, the medium was changed to DH2 medium containing 2% horse serum in DMEM supplemented with P/S (1x) with MβCD (the solvent, sterile water, was added for control) at a concentration of 1 mM. After induction of differentiation in the DH2 medium for 1 day with or without MβCD, the cultured chick myogenic cells were further analyzed. All animal experiments were performed following ethical guidelines set by Kindai University, and animal protocols were reviewed and approved by the Kindai University Animal Care and Use Committee.

### Immunofluorescence

For immunofluorescence studies, cells grown on a 4-well plate were fixed with freshly prepared 4% paraformaldehyde in PBS for 15 min at room temperature and permeabilized and blocked with 1% Triton-X, 1% BSA, and 10% FBS-contained PBS for 1 h at room temperature. The cells were incubated with primary antibodies in 10% FBS, 1% BSA, and 1% Triton-X-containing PBS overnight at 4°C. On the following day, the cells were washed three times in 0.1% Triton/PBS and incubated with fluorescent-labeled IgG (H + L) secondary antibodies (Abcam, Goat anti-mouse (Alexa Fluor 488): ab150113, (Alexa Fluor 594): ab150116, Goat anti-rabbit (Alexa Fluor 594: ab150080) at 1:500 dilutions in 1% FBS, 1% BSA, and 1% Triton-X-containing PBS for 2 h at room temperature. The cells were washed three times and filled with 0.1% Triton-containing PBS, and nuclei were counter-stained with DAPI (Sigma-Aldrich, D9542) for immunofluorescence. Primary antibodies used in this study include Anti-Myosin Heavy Chain antibody (MF20) (1:500, R&D Systems, MAB4470), Anti-Myogenin antibody (1:500, Abcam, ab124800), Anti-p-Histone H3 antibody (C-2) (1:1000, Santa Cruz, sc-374669), and Anti-Cleaved Caspase-3 antibody (Asp175) (5A1E) (1:500, Cell Signaling Technology, #9664). The observation was performed under a microscope (Keyence, BZ-X710).

### RNA preparation and real-time PCR

Total RNAs were extracted using RNeasy Mini Kit (QIAGEN, 74104) according to the manufacturer’s instructions. RNAs were reverse-transcribed using ReverTra Ace qPCR RT Master Mix (TOYOBO, FSQ-201), and real-time PCR was performed using THUNDERBIRD SYBR qPCR Mix (TOYOBO, QPS-201) in MIC qPCR (bio molecular systems). Expression levels of each gene were normalized according to each purpose to Gapdh or Hprt expression and calculated using the comparative CT method. The primer sequences are shown below: Gapdh-F (AGG​TCG​GTG​TGA​ACG​GAT​TTG), Gapdh-R (TGT​AGA​CCA​TGT​AGT​TGA​GGT​CA), Hprt-F (CAG​TCC​CAG​CGT​CGT​GAT​TA), Hprt-R (AGC​AAG​TCT​TTC​AGT​CCT​GTC), MyoD-F (GAT​GGC​ATG​ATG​GAT​TAC​AGC​GGC), MyoD-R (GTG​GAG​ATG​CGC​TCC​ACT​ATG​CTG), Myogenin-F (CCC​TAT​TTC​TAC​CAG​GAG​CCC​CAC), Myogenin-R (GCG​CAG​GAT​CTC​CAC​TTT​AGG​CAG), Myh7-F (ATG​CTG​ACA​GAT​CGG​GAG​AA), Myh7-R (GGT​TGG​CTT​GGA​TGA​TTT​GA), Mymk-F (CCT​GCT​GTC​TCT​CCC​AAG), Mymk-R (AGA​ACC​AGT​GGG​TCC​CTA​A), Srebf2-F (ACC​TAG​ACC​TCG​CCA​AAG​GT), Srebf2-R (GCA​CGG​ATA​AGC​AGG​TTT​GT), Hmgcs1-F (GCC​GTG​AAC​TGG​GTC​GAA), Hmgcs1-R (GCA​TAT​ATA​GCA​ATG​TCT​CCT), Hmgcr-F (CTT​GTG​GAA​TGC​CTT​GTG​ATT​G), Hmgcr-R (AGC​CGA​AGC​AGC​ACA​TGA​T), Pmvk-F (AAA​ATC​CGG​GAA​GGA​CTT​CGT), Pmvk-R (AGA​GCA​CAG​ATG​TTA​CCT​CCA), Cyp51-F (GAC​AGG​AGG​CAA​CTT​GCT​TTC), Cyp51-R (GTG​GAC​TTT​TCG​CTC​CAG​C).

### Modulation of cholesterol

For modulating the amount of membrane cholesterol, solubilized cholesterol (Sigma, C8667) (as a complex of MβCD and cholesterol in EtOH) was added at 15 µM (the solvent, EtOH, was added for control) as an optimized concentration (Supplementary Figure S4) in the induction of differentiation of C2C12 cells.

### Qualitative estimation of membrane cholesterol

Cells were grown on rounded cover glass (MATSUNAMI, 83-0232) treated with Poly-L-lysine coating solution (COSMO BIO, SPL01) in a 4-well plate and fixed with 4% paraformaldehyde in PBS for 15 min at room temperature to label membrane cholesterol. The cells were then washed three times in PBS and incubated with 1.5 mg/ml glycine in PBS for 10 min at room temperature to quench the paraformaldehyde. After that, cells were stained with filipin working solution (0.05 mg/ml filipin complex (Sigma-Aldrich, F9765) in 10% FBS-contained PBS) for 2 h at room temperature and washed three times in PBS. Finally, images were collected using a confocal microscope (Olympus, FV-3000).

### Flow cytometry

Cells were disassociated using 0.25% Trypsin/EDTA (nacalai tesque, 35554-64), washed with 1% BSA/PBS, and fixed in 1% PFA on ice for 1 h. After washing with 1% BSA/PBS, the cells were permeabilized/blocked with 1% Triton-X-, 1% BSA- and 10% FBS-contained PBS for 1.5 h at room temperature. They were then incubated overnight at 4°C with appropriate amounts of primary antibodies, including the Anti-p-Histone H3 antibody (C-2) (1:100, Santa Cruz, sc-374669) and Mouse mAb IgG_2b_ Isotype Control monoclonal antibody (1:100, Santa Cruz, sc-3879) in 1% FBS-, 1% BSA- and 0.1% Triton-X-contained PBS. On the following day, after washing with 0.1% Triton/PBS, the cells were incubated with fluorescent-labeled IgG (H + L) secondary antibodies (Abcam, Goat anti-mouse, Alexa Fluor 594) at 1:500 dilution in 1% FBS-, 1% BSA- and 0.1% Triton-X-contained PBS for 2 h at room temperature. Immediately after washing with 0.1% Triton/PBS, the cells were resuspended in an ice-cold Sheath fluid (Sysmex, CG-974-836) at 1 × 10^6^ cells/mL and analyzed on a flow cytometer. FACS analysis was performed using Sysmex RF-500 with FlowJo (BD Biosciences), and a minimum of 10,000 events were recorded for each sample. Fluorescence minus control was used to gate the subpopulations.

### RNA-sequence and data analysis

Total RNAs were extracted using the RNeasy Mini Kit (QIAGEN, 74104) following the manufacturer’s instructions. For each experiment, two independent biological replicates were used for transcriptomic analysis. Sequencing libraries were prepared using the NEBNext Ultra II Directional RNA Library Prep Kit (NEB) and were sequenced on an Illumina Novaseq platform to generate 150 bp paired-end reads. The adaptors of the obtained raw sequence data were trimmed using Trim Galore (v0.6.3), and quality control using FastQC (v0.11.7). Processed reads were aligned to the mouse reference genome (UCSC mm10) using STAR (v2.7.0c) (Dobin et al., 2013). FeatureCounts v1.5.2 was used to generate the read-counting data for each gene. Differential gene expression analysis was performed using TCC (Sun et al., 2013), (Tang et al., 2015). PCA analysis and Heatmap clustering were performed using the princomp, and heatmap packages from R. Gene Ontology (GO) analysis was performed using the clusterProfiler (v4.4.4) package from R (Yu et al., 2012).

### Statistical analysis

Statistical analysis was performed using one-way ANOVA, when significant, group differences were evaluated using a Student’s t-test. *p* values <0.05 were statistically significant.

## Results

### Inhibition of myoblast differentiation with MβCD in C2C12 cells

To inhibit myoblast differentiation, we examined MβCD, a derivative of parent β-cyclodextrin, as a potential component (Gonzalez Pereira et al., 2021), (Mortensen et al., 2016). MβCD was selected because it has been shown to be the most effective agent among the various agents used for cholesterol depletion from cells (Gotoh et al., 2014) as well as a food additive. It is a widely accepted fact that when cells are depleted of cholesterol, they lose their ability to fuse myoblasts, and also re-introduction of cholesterol to the culture medium can restore it, as demonstrated in previous studies (Mukai et al., 2009), (Taulet et al., 2009). The addition of MβCD to C2C12 cells cultured under serum-reduced conditions resulted in a significant reduction in the appearance of elongated cells, a characteristic of differentiating myotubes, compared to the control group where elongated cells were evident (Figure 1B, indicated by white arrowheads). To investigate the impact of MβCD on myogenic differentiation, we evaluated the expression of key transcription and protein markers of myogenic differentiation (Figures 1C, D). The results of quantitative PCR analysis and immunofluorescence showed that cells cultured under MβCD-containing conditions had significantly lower expression levels of myotube differentiation markers compared to the control group (Figures 1C, D). Furthermore, the inhibitory effect of MβCD on myotube differentiation was observed in a dose-dependent manner (Supplementary Figure S1). These findings suggest that MβCD can inhibit myotube differentiation in C2C12 cells under serum-reduced conditions.

### Arrest of differentiation by MβCD at MyoD-positive stage in C2C12 cells

**FIGURE 2:**
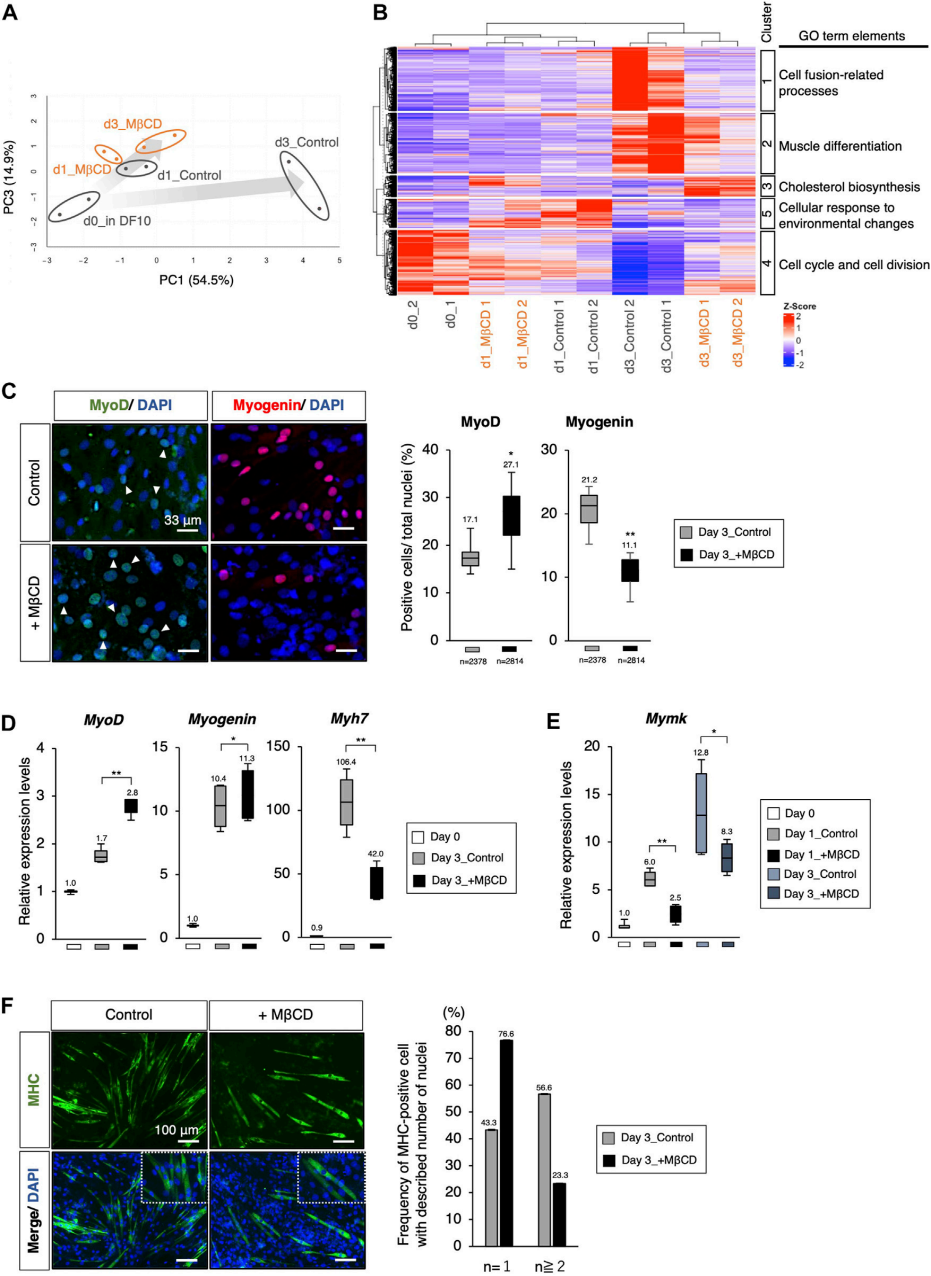
The Arrest of myoblast differentiation by MβCD at MyoD-positive stage. **(A, B)** Bioinformatic analysis of RNA-sequence data with C2C12 cells cultured in each condition. **(A)** The principal component analysis (PCA) with differentially expressed genes (DEGs) visualizing the differentiation arrest of C2C12 cells with MβCD. The five groups include d0_in DF10 (C2C12 cells cultured in DF10 before differentiation induction, black), d1_Control (differentiation in DH2 medium for 1 day, black), d3_Control (for 3 days, black), d1_MβCD (differentiation in DH2 medium with MβCD for 1 day, orange), and d3_MβCD (with MβCD for 3 days, orange). Replicates of the same conditions are surrounded by an ellipse. The extent of differentiation progression in control and with MβCD is represented by arrows colored with gray gradients. **(B)** A hierarchical clustering heatmap of Z-scaled counting data of DEGs into five different cultured conditions shown in Figure 2A. The heatmap illustrates Z-scaled expression levels of those genes. Cluster 1–5 is categorized as the group of molecules associated with the Gene Ontology Biological Processes (GO_BP), and the predicted annotations for each cluster are indicated. The results of GO_BP analysis in each cluster are shown in Supplementary Figure S2. **(C)** Immunofluorescence for MyoD- and Myogenin-protein expression confirms early- and mid-differentiation of myoblasts respectively in C2C12 cells on day 3 with DH2 medium compared with and without MβCD (3 mM). Representative images are shown on the left, where white arrowheads indicate MyoD-positive cells. The frequency of MyoD- and Myogenin-positive cells was calculated as a percentage of total nuclei. The total number of counted cells is indicated in the graph. **(D)** Quantitative PCR analysis of the expression of muscle markers in growing C2C12 cells cultured in DF10 (Day 0), differentiating C2C12 cells cultured in DH2 (Day3_control), and with MβCD (Day3_+ MβCD). Error bars indicate s.d. (n = 4, biological replicates). **(E)** Quantitative PCR analysis of expression of Myomaker (Mymk, myoblast fusion factor). Error bars indicate s.d. (n = 4, biological replicates). **(F)** Immunofluorescence for MHC (Myosin Heavy Chain) protein expression on day 3 with DH2 medium compared with and without MβCD (3 mM). Multi- and single-nucleated myotubes at higher magnification are represented by square dot lines in the bottom images. The number of nuclei per MHC-positive cell was indicated in the graph with the number of measured cells (n = 60 in each condition). Nuclei were counterstained with DAPI. Scale bar, 100 μm. The one-way ANOVA followed by the t-test with **p < 0.01, *p < 0.05.

We conducted a gene profiling analysis using RNA-sequence to evaluate the inhibitory effects of MβCD on differentiation (Figures 2A, B). Our results from the PCA analysis indicated that C2C12 myoblast cells differentiate into myotubes with DH2 medium and PC1 represents the extent of differentiation (Figure 2A). Interestingly, the plots of day 3 in differentiation with MβCD were positioned close to day 1 plots, indicating that MβCD has potent inhibitory effects on differentiation. We used hierarchical clustering heatmap analysis to categorize the group of molecules associated with GO_BP into five clusters based on the differentially expressed genes (DEGs) (Figure 2B). At day 3 differentiation, the expression of molecules associated with cell fusion-related processes genes, lipid metabolism, peptidases, and other proteolytic molecules, and molecules related to extracellular matrix and structure were significantly reduced in MβCD-contained conditions (Cluster 1). Additionally, the expression of muscle differentiation-related genes, such as muscle differentiation markers and molecules related to mitochondrial respiration, was significantly reduced (Cluster 2). The effect of cholesterol depletion of MβCD was evident in the cellular response, with a marked increase in cholesterol biosynthesis-related molecules from day 1 after the addition of MβCD (d1_MβCD and d3_MβCD) (Cluster 3). Notably, the high expression of cell cycle- and cell division-related molecules seen before differentiation induction was markedly reduced immediately after differentiation induction (d1_Control & d1_MβCD) and almost absent by day 3 under control conditions, whereas a certain amount of expression could still be seen even on day 3 of differentiation with MβCD (d3_MβCD) (Cluster 4) (Figure 2B).

While C2C12 cells are commonly used as a model system to study skeletal muscle differentiation, they have also been shown to have multipotential capabilities such as osteogenesis and adipogenesis by specific stimuli (Katagiri et al., 1994), (Teboul et al., 1995), leading to a possibility that they differentiate into those cell types other than myogenesis in response to MβCD-treatment. However, no changes in gene expression calculated from RNA-seq data were detected that would support that possibility (Supplementary Figure S3).

Through PCA analysis, it was observed that there was no apparent difference between the control and MβCD-treated cells on day 1 differentiation, but a significant difference was observed on day 3. The control cells exhibited advanced muscle differentiation, while the MβCD-treated cells remained at a stage similar to day 1 differentiation, suggesting that the addition of MβCD inhibits the early stages of differentiation (Figures 2A, B). To determine at what stage MβCD inhibits the differentiation of C2C12 cells, the expression of various muscle differentiation markers was analyzed. It was found that the percentage of MyoD-positive cells, an early marker of muscle differentiation, was significantly increased in the MβCD-contained condition compared to the control on day 3 after differentiation induction. In contrast, the percentage of Myogenin-positive cells, a mid-to-late-stage marker, was significantly decreased. Moreover, transcriptional analysis also revealed that the differentiation progression was arrested at the MyoD-positive stage (Figures 2C, D).

Given the fact that cell fusion between myoblasts accompanies their differentiation process, the effect of MβCD on myoblast cell fusion was investigated. Considering that cell fusion of myoblasts is observed after 1 day of differentiation in C2C12 cells induced by the DH2 condition, it was hypothesized that MβCD might have inhibitory effects on myoblast cell fusion. Myomaker (Mymk), a Myoblast-specific molecule that plays a crucial role in cell fusion between Myoblasts, was studied (Bi et al., 2017). The results of quantitative PCR analysis demonstrated that *Mymk* expression was mainly downregulated in MβCD-treated conditions (Figure 2E). Correspondingly, MHC-positive cells with a single nucleus were predominant in the MβCD-treated conditions (Figure 2F). These observations indicate that MβCD inhibits the progression of C2C12 cell differentiation at the MyoD-positive stage of muscle differentiation, and part of this inhibitory effect is due to its interference with cell fusion between myoblasts.

### Depletion of membrane cholesterol required for myotube differentiation by MβCD

**FIGURE 3:**
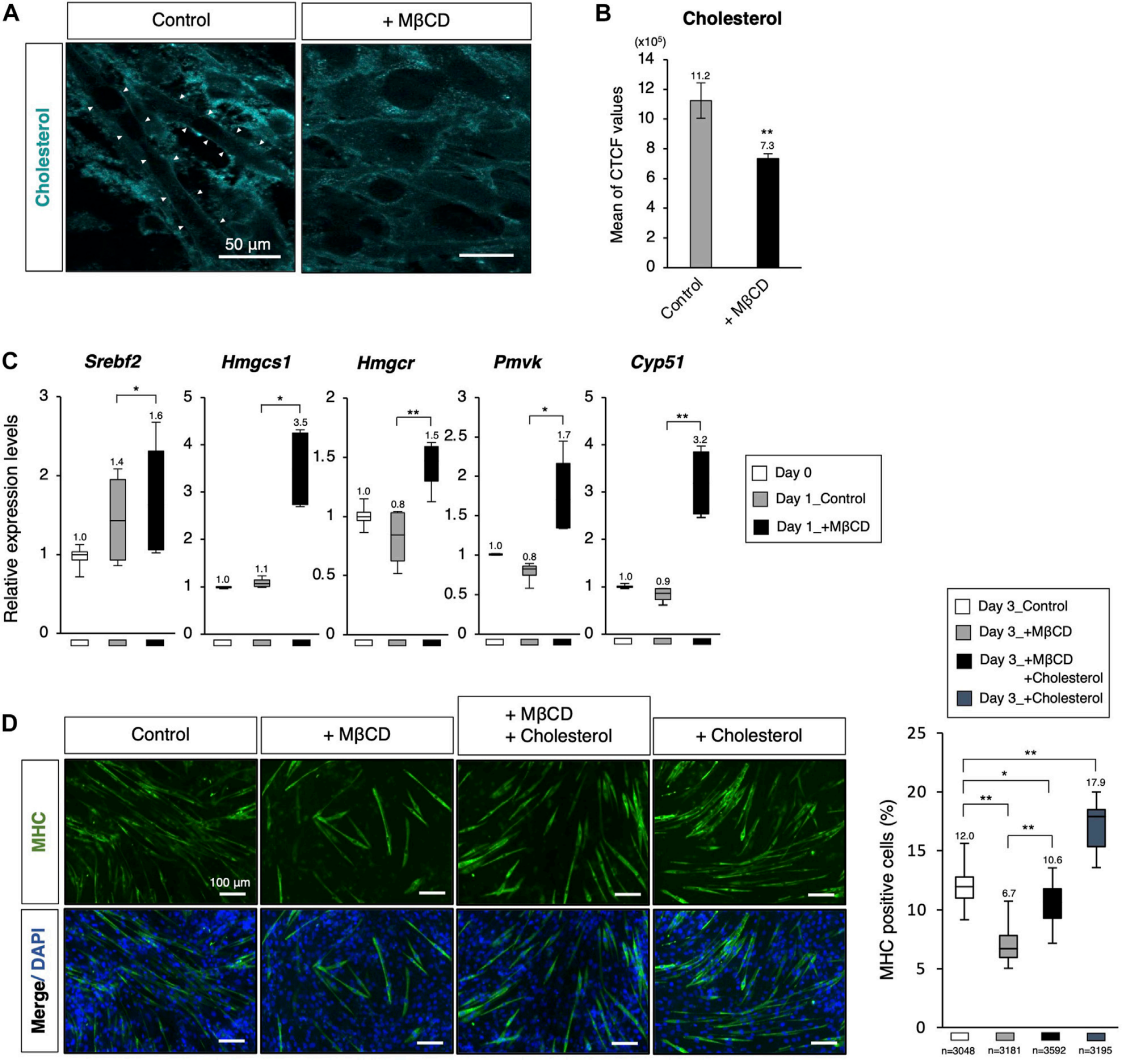
Decreasing membrane cholesterol required for myotube differentiation by MβCD. **(A)** Filipin fluorescence staining for membrane cholesterol in C2C12 cells cultured for 44 h. Linearized signals of cholesterol-enriched membranes were observed on elongated cells, which are morphologically differentiating into myotubes. The images for filipin staining were obtained under the same exposure time for all conditions, and white arrowheads were used to indicate these signals. The scale bar was set at 50 μm. **(B)** Attenuated cellular cholesterol level under MβCD-treated conditions determined by measuring cell fluorescence with filipin staining performed after 24 h of induction of differentiation under DH2 conditions, and the CTCF value was calculated using ImageJ from fluorescence microscopy images (n = 6). **(C)** Quantitative PCR analysis of expression of genes related to *de novo* cholesterol biosynthesis with growing C2C12 cells cultured in DF10 (Day 0), differentiating C2C12 cells cultured in DH2 for 1 day (Day1_Control), and those treated with MβCD (Day1_+ MβCD). Error bars indicate s.d. (n = 4, biological replicates). **(D)** Rescued and enhanced differentiation into myotubes by modulation of the cellular cholesterol level. Immunofluorescence for MHC protein expression in C2C12 cells on day 3 of induction in DH2 medium with MβCD (1 mM) or cholesterol (15 µM). Nuclei were counterstained with DAPI. The frequency of MHC-positive cells was calculated as a percentage of total nuclei. The total number of counted cells is indicated in the graph. The scale bar was set at 100 μm. The error bars represent s.d. and The one-way ANOVA followed by the t-tests with *p < 0.05 and **p < 0.01.

To evaluate the effectiveness of MβCD in reducing membrane cholesterol levels in differentiating C2C12 myoblast cells, filipin fluorescence staining was utilized to label cholesterol. We observed reduced cholesterol signals in the membrane of C2C12 myoblast cells cultured in DH2 medium for 44 h with MβCD treatment compared to the control (Figures 3A, B). Furthermore, intense linearized signals of membrane cholesterol were found in cells exhibiting elongated cell morphology, which is a characteristic of myotubes (white arrowheads in Figure 3A). Consistent with gene profiling analysis using RNA-sequence (Figure 2B), we observed significant upregulation of cholesterol biosynthesis-related genes in cells cultured with MβCD for 1 day, which is the primary response of cholesterol-depleted cells to compensate, while no change was observed in the control compared to cells cultured in DF10 as day 0 (Figure 3C). To investigate the possibility that MβCD-induced cellular cholesterol depletion inhibits C2C12 myoblast cell differentiation, we performed rescue experiments by administering cholesterol for myotube differentiation (Figure 3D). The optimized concentration of cholesterol solution for myotube differentiation of C2C12 myoblast cells without cytotoxicity was determined to be 15 µM (Supplementary Figure S4). Immunofluorescence for MHC demonstrated that the inhibitory effect of MβCD on myotube differentiation was significantly compensated by the administration of cholesterol (Figure 3D). Additionally, administration of cholesterol alone was found to increase differentiation efficiency compared to the control. These results suggest that cellular cholesterol in the plasma membrane of C2C12 myoblast cells is essential for differentiation into myotubes and that MβCD-induced reduction of cholesterol levels causes inhibition of myotube differentiation in serum-reduced conditions.

### Involvement of cellular cholesterol in apoptotic cell death of myoblasts

**FIGURE 4:**
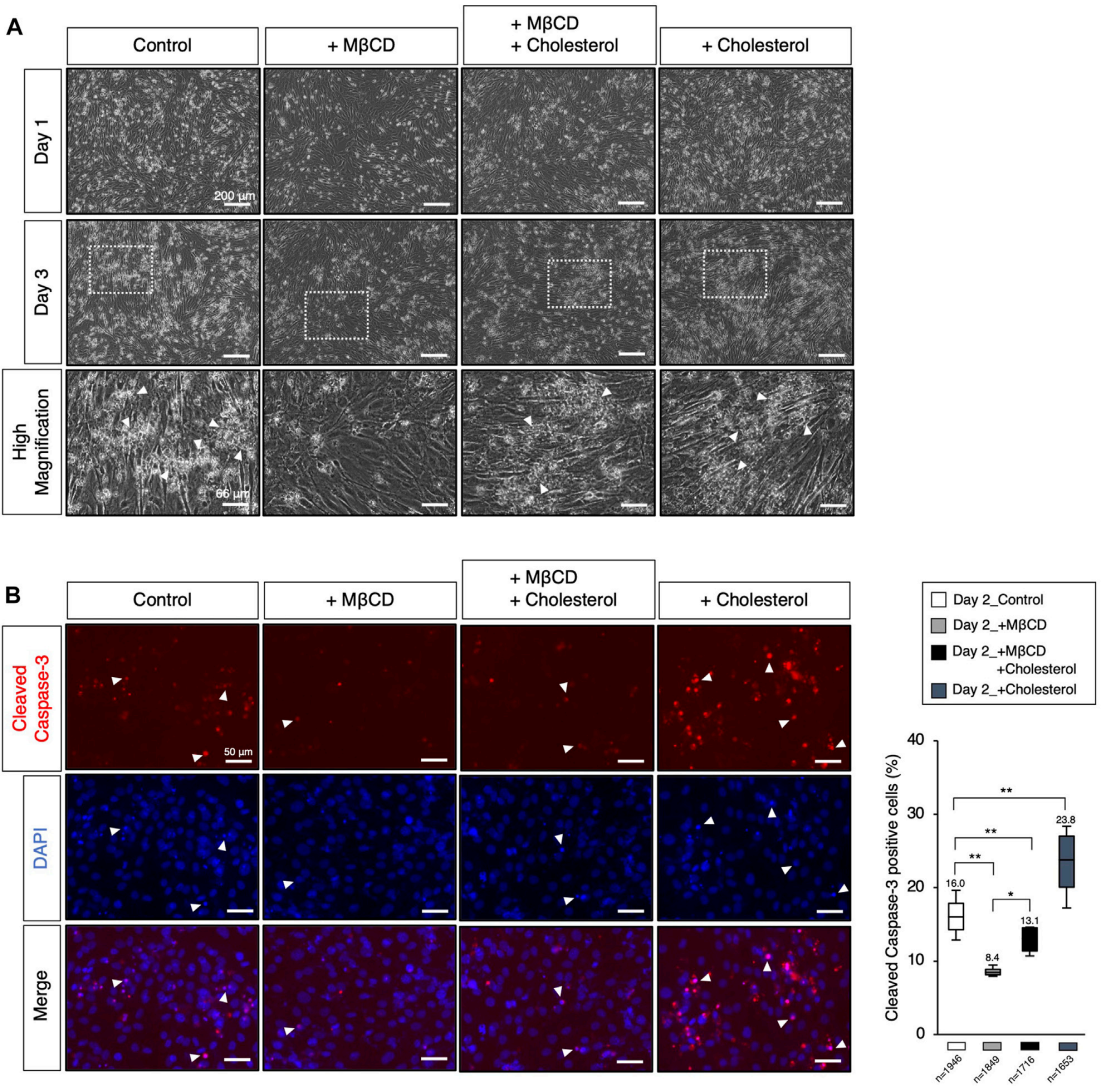
Modulation of the cellular cholesterol level affects apoptotic cell death. **(A)** The representative images of cultured C2C12 cells under DH2 culture conditions, with the modulation of cholesterol levels using cholesterol (15 µM) and MβCD (3 mM). The images at the bottom correspond to square dot lines in the middle images at higher magnification. White arrowheads indicate representative cell death. Reduced cell death under MβCD-contained conditions was abolished by adding cholesterol. The scale bar for the top and middle images is 200 μm, and for the bottom images is 66 μm. **(B)** Representative images for cleaved Caspase-3 protein expression, a definitive marker of apoptotic cell death, on day 2 of myoblast differentiation. White arrowheads indicate representative cleaved Caspase-3-positive cells and prominent nuclear condensation by DAPI. The scale bar is 50 μm. The frequency of cleaved Caspase-3-positive cells was calculated as a percentage of total nuclei. The total number of counted cells is indicated in the graph. Error bars indicate s.d. and the one-way ANOVA followed by the t-tests with *p < 0.05 and **p < 0.01.

Apoptotic cell death has been reported to play a role in promoting myoblast fusion through contact-dependent signaling with neighboring cells to encourage fusion among healthy differentiating myoblasts (Hochreiter-Hufford et al., 2013). During our observations of myotube differentiation with MβCD, we discovered that cell death of myoblasts, which consistently occurs in serum-reduced medium conditions, was significantly reduced in MβCD-contained conditions (Figure 4A). This led us to hypothesize that the depletion of cellular cholesterol by MβCD could potently block the induction of cholesterol-dependent apoptotic cell death, impair myoblast fusion, and consequently hinder myotube differentiation. We carefully observed the appearance of dead cells (Figure 4A) and validated apoptotic cell death by immunofluorescence for cleaved Caspase-3 (Figure 4B). These results suggest that MβCD effectively blocks apoptotic cell death, and cholesterol significantly restores it. Furthermore, cholesterol alone significantly induced cleaved Caspase-3-positive cells (Figure 4B). Thus, the blockage of apoptotic cell death in C2C12 myoblast cells during differentiation caused by depletion of cholesterol with MβCD is one of the potential mechanisms by which MβCD inhibits myotube differentiation in C2C12 cells since dead cells by apoptosis promote adjacent myoblasts to differentiate into myotubes by inducing myoblast cell fusion (Hochreiter-Hufford et al., 2013).

### The involvement of MβCD in myoblast proliferative capacity persists even under serum-reduced conditions

**FIGURE 5:**
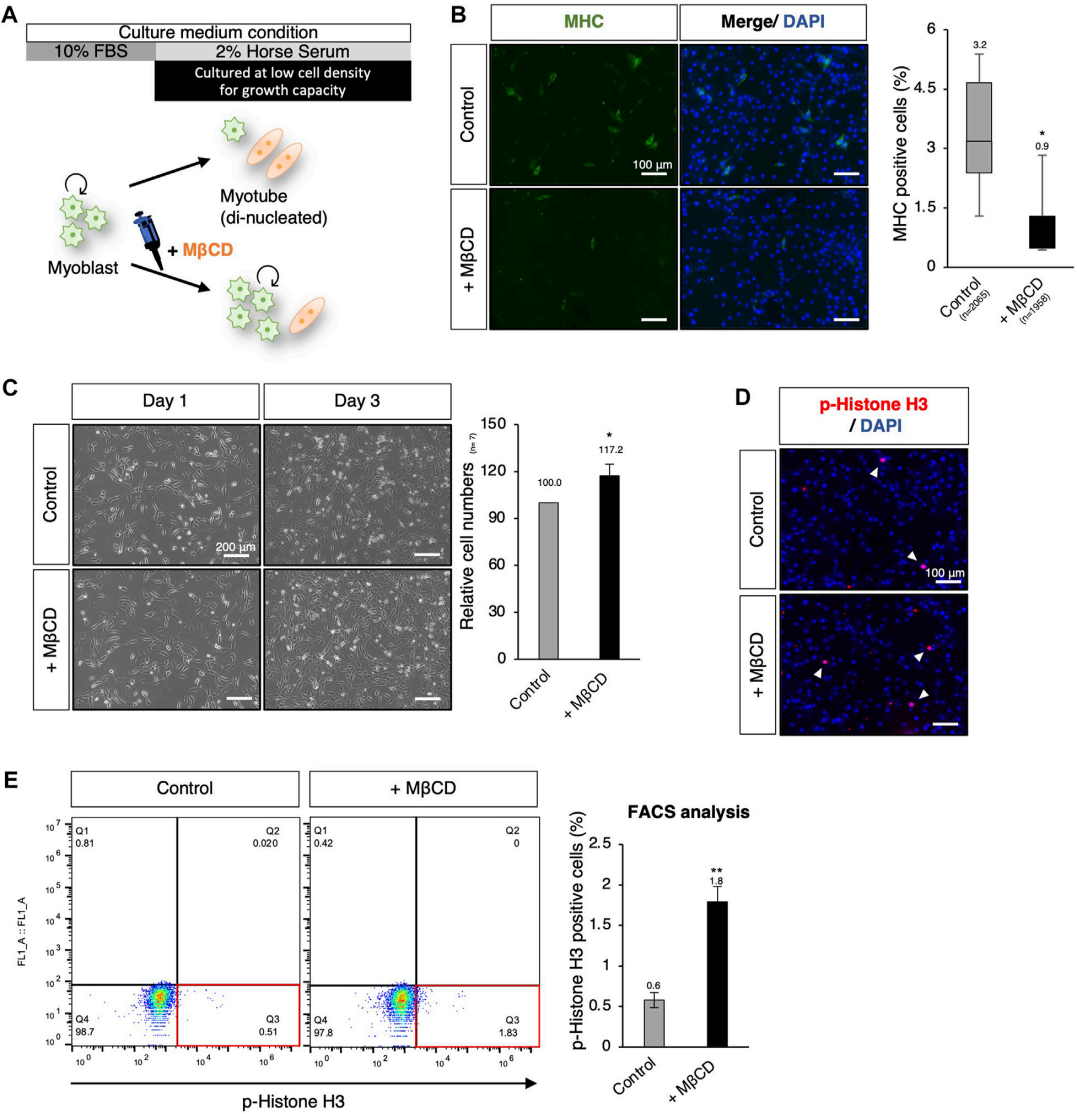
MβCD involves the extended proliferative capacity of myoblasts even under conditions that induce differentiation. **(A)** Schematic representation of the experimental workflow for inducing myogenic differentiation in C2C12 myoblast cells at low cell density. To evaluate the effect of MβCD on cell proliferation under DH2 medium conditions, C2C12 myoblast cells were cultured at a low density of 40%–50% confluency before induction of differentiation. **(B)** Representative images of immunofluorescence for MHC protein expression in C2C12 cells on day 3 of induction with DH2 medium. The frequency of MHC-positive cells was calculated as a percentage of total nuclei. **(C)** The relative cell number of C2C12 cells cultured with MβCD under DH2 conditions at low cell density. Representative images of C2C12 cells grown under DH2 conditions at low cell density were captured and the cell number on day 3 was counted. Error bars indicate s.d. (n = 7, biological replicates) **(D)** Representative images of immunofluorescence for phospho-Histone H3 (p-Histone H3) protein expression (a marker of mitosis) in C2C12 cells on day 3 of induction with DH2 medium. Nuclei were counterstained with DAPI, and white arrowheads indicate representative p-Histone H3-positive cells. **(E)** FACS analysis for the effect of MβCD on p-Histone H3-positive cells in C2C12 cells cultured at low cell density on day 3 of induction with DH2 medium. The proportion of p-Histone H3-positive cells in total cell number was calculated as a percentage, and the results were analyzed using FlowJo. Error bars indicate s.d. (n = 3, biological replicates) and the one-way ANOVA followed by the t-tests with *p < 0.05 and **p < 0.01.

Using hierarchical clustering heatmap analysis, we observed higher expression of cell cycle-related genes in cells treated with MβCD compared to the control (Figure 2B). Thus, we investigated the effects of MβCD on cell proliferative capacity in serum-reduced conditions by preparing C2C12 myoblast cells at low density (40%–50% confluency) for further cell growth capacity, as high cell density (90%–100% confluency) is typically required for efficient differentiation (Figure 5A). We first determined whether MβCD could have a comparable inhibitory effect on the differentiation of C2C12 myoblast cells at low cell density (Figure 5B) as it does at high cell density (Figure 1). The optimized concentration of MβCD was found to be 0.8 mM without cytotoxicity at low cell density (Supplementary Figure S5). After 3 days of cell growth under serum-reduced conditions, we observed that not only the relative number of cells but also the frequency of cells positive for phosphorylated histone H3, a mitosis marker, significantly increased in MβCD-treated conditions compared to the control (Figures 5C–E). These “mitogenic” effects of MβCD on C2C12 myoblast cells were only observed under serum-reduced conditions of DH2, but not under serum-rich conditions of DF10 (Supplementary Figure S6), which strongly supports our assumption that the mitogenic effect of MβCD on C2C12 myoblast cells is due to its inhibitory effect on myoblast differentiation into myotubes, rather than directly enhancing cell proliferation of myoblasts.

### Ex vivo analysis of MβCD competence using myogenic cells derived from chick embryos

**FIGURE 6:**
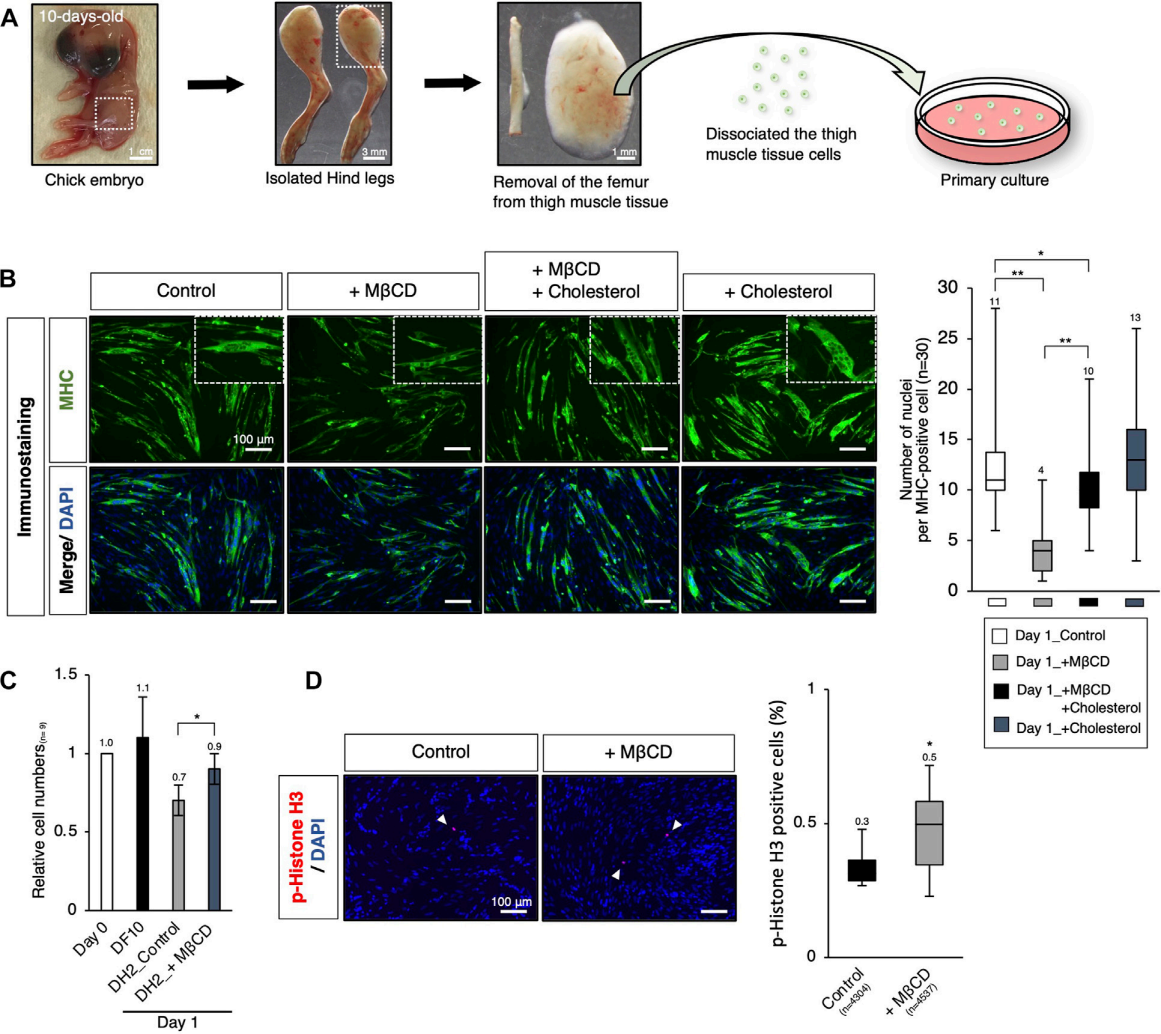
*Ex vivo* analysis of MβCD competence in inhibiting myotube differentiation and involvement in myogenic proliferation in chick embryos. **(A)** Schematic representation of the experimental workflow for primary culture of embryonic chick myogenic cells. Surgical dissection was used to prepare isolated chick thigh muscle cells without the femurs obtained from 10-day-old chick embryos. The thigh muscle tissues in a hind leg are indicated by square dot lines in the images. **(B)** Representative images of immunofluorescence for MHC protein expression in primary cultured chick thigh muscle cells on day 1 with DH2 medium. Differentiating multi-nucleated myotubes are indicated by square dot lines. Nuclei were counterstained with DAPI. The number of nuclei per MHC-positive cell, an indicator of the extent of differentiation as myotube, was counted. **(C)** The relative cell number of chick myogenic cells cultured with MβCD. Dissociated thigh muscle cells were plated on a gelatin-coated well and cultured in a DF10 medium for 1 day to ensure cell attachment. The cells cultured for 1 day in a DF10 medium were considered “Day 0”. The cells were then cultured in the indicated conditions for 1 day. Error bars represent s. d. (n = 7, biological replicates). **(D)** Immunofluorescence images for phospho-Histone H3 (p-Histone H3) protein expression in cultured chick myogenic cells on day 1 with DH2 medium. Nuclei were counterstained with DAPI. Representative p-Histone H3-positive cells are indicated by white arrowheads. The frequency of p-Histone H3-positive cells was calculated as a percentage of total nuclei. The total number of counted cells is indicated in the graph. Error bars represent s.d. (n = 3, biological replicates) and the one-way ANOVA followed by t-tests with *p < 0.05 and **p < 0.01.

To enable the industrialization of cultured meat production, it is important to investigate the potential of MβCD in livestock animals through *ex vivo* analysis. Previous studies have shown that short-term treatment with MβCD for 30 min in primary chick myogenic cells promotes myoblast proliferation (Portilho et al., 2012), but its long-term effects on proliferation and differentiation inhibition have not been established. In our experiments, primary cultures of chick myogenic cells were used to assess MβCD competence, as seen in the C2C12 myoblast cells mentioned above. The chick thigh muscle cells were obtained from 10-day-old chick embryos and plated. On day 1 under serum-reduced conditions, we evaluated the effects of MβCD on myotube differentiation and cell growth. The addition of MβCD resulted in the blocked maturation of MHC-positive myotubes with a narrower width and a smaller number of nuclei per cell (Figure 6B). However, MβCD led to an increase in the number of cells under serum-reduced conditions (Figure 6C), due to its ability to enhance cell proliferative capacity, as evidenced by the increased number of p-Histone H3-positive cells (Figure 6D).

## Discussion

The establishment of safe and cost-effective cell culture methods using serum-free media is essential for the future commercial viability and acceptance of cultured meat (van der Valk et al., 2010). The results of this study, which showed that the use of MβCD can inhibit muscle cell differentiation and maintain proliferative potential, provide significant insight into achieving this goal under serum-free and serum-reduced conditions, where myoblasts differentiate rapidly and cease to proliferate. Albeit only as a first step toward the goal, the results support our contention that the use of safe food additives to inhibit myoblast differentiation could be an effective approach toward establishing serum- and growth factor-independent myocyte proliferation techniques.

**FIGURE 7:**
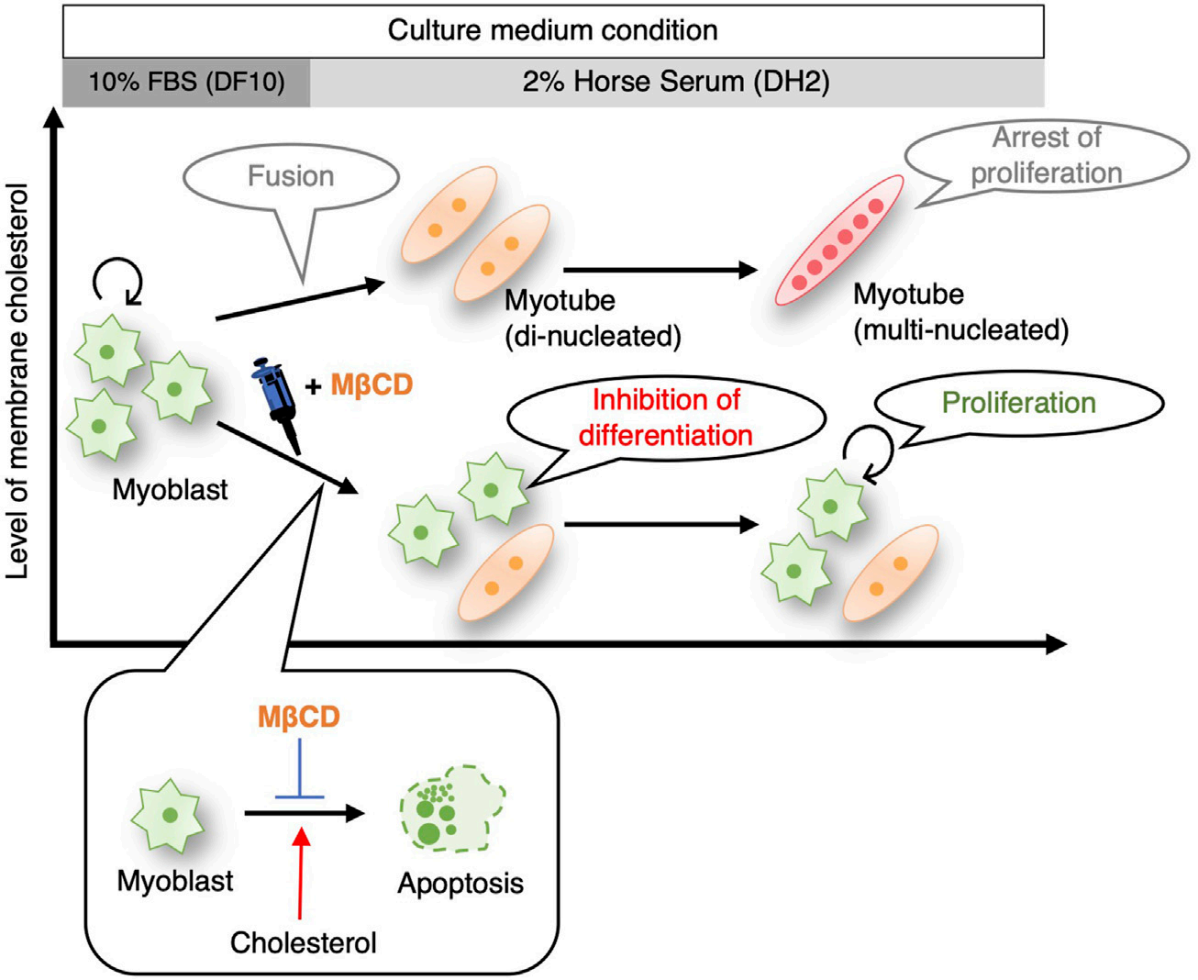
Schematic illustrating summarizing that inhibition of myoblast differentiation into myotube with MβCD ensures proliferation of myogenic cells in serum-reduced culture conditions. MβCD is a potent inhibitor of myoblast differentiation into myotubes by reducing cellular cholesterol levels in myoblasts. This enables the continuous proliferation of myoblasts even under serum-depleted conditions. By lowering cellular cholesterol levels, MβCD inhibits apoptotic cell death, which is necessary for myogenic differentiation, and this is one of the effective mechanisms by which MβCD functions as a myoblast differentiation inhibitor under serum-reduced conditions.

While pluripotent stem cells such as human iPS cells and mouse ES cells have been successfully cultured without serum (Lu et al., 2006), (De Los Angeles et al., 2019), (Ying et al., 2003), the maintenance of their undifferentiated and proliferative potential still depends on the addition of growth factors and chemical compounds. Although cancer cells have been reported to have proliferative potential under serum-free culture in absence of growth factors (Wu and Näär, 2019), (Takii et al., 2022), this has been limited to specific cell types and culture periods. In our study, we observed that the addition of MβCD to the culture medium inhibited the differentiation of C2C12 mouse skeletal myoblasts into myotubes (Figure 1), and maintained their proliferative capacity even under serum-reduced conditions in absence of growth factors (Figures 5, 7). These results were replicated in muscle cells from chick embryos (Figure 6), indicating that the inhibitory effect of MβCD on differentiation is independent of animal species. These findings offer the potential to expand myoblasts using serum-free media for the future production of cultured meat from certain livestock animals.

In the case of primary cultured muscle cells from 11-day-old chick embryos, however, conflicting results have been reported that MβCD plays a role in promoting myoblast fusion and further differentiation (Mermelstein et al., 2005), (Possidonio et al., 2014). In these analyses, primary cultured muscle cells were treated with 2 mM MβCD for only 30 min and subsequently evaluated at 24, 48, and 72-h intervals under withdrawal of MβCD. The “transient cholesterol removal treatment” is anticipated to stimulate the cholesterol biosynthetic system as a subsequent cellular response, and it cannot be ruled out that intracellular cholesterol may be elevated during the process of continued culture following MβCD withdrawal, as has been observed in cancer cells (Zidovetzki and Levitan, 2007) and T lymphocyte (Fülöp et al., 2001). Although activation of the cholesterol biosynthesis system by MβCD treatment was also observed in the present experiment (Figure 3C), the decrease in plasma membrane cholesterol by Filipin staining indicates that there is no such concern under the experimental conditions of continuous MβCD addition (Figures 3A, B). Furthermore, another research group recently presented results on the effects of 96-h continuous treatment of MβCD during induction of C2C12 cell differentiation. The data revealed that depletion of cholesterol from the cell membrane caused by MβCD increases membrane tension, which in turn obstructs membrane fusion and ultimately prevents multinucleation during myogenic differentiation (Chakraborty et al., 2022). Despite the differences in the explanation of the mechanism, these findings provide robust evidence in support of our assertion that continuous treatment of MβCD inhibits the differentiation of myogenesis.

The results of PCA analysis using RNA-sequence and q-PCR analysis (Figure 2) indicated that the addition of MβCD did not maintain C2C12 cells in an undifferentiated state as actively growing myoblasts, but rather inhibited further differentiation at an early stage, where they express high levels of MyoD. The absence of increased expression of Myomixer (Bi et al., 2017) with MβCD suggested that many mononuclear myoblasts were in the pre-stage of cell fusion and that further differentiation was halted (Figure 2). MyoD as expressed in proliferating myoblasts is known to allow myoblast proliferation (Weintraub, 1993) and its increased expression is associated not only with cell cycle exit with the onset of differentiation, but also with cell cycle in G2/M phase (Kitzmann et al., 1998). Thus, the maintained proliferative potential with MβCD is not contradicted by the presence of a significant number of MyoD-expressing cells (Figures 2C, D).

In this study, we postulate that the impact of MβCD is the inhibition of differentiation, as opposed to just a delay in differentiation. This supposition is based on the significant reduction in apoptotic cell death of myoblasts following MβCD addition (Figure 4). Furthermore, our findings that cholesterol-dependent cell death occurs in C2C12 cells (Figure 4), coupled with the cholesterol-depleting effect of MβCD, indicate that MβCD ‘s effect on differentiation is due to its inhibition, rather than delay.

We are currently exploring four possible mechanisms that may explain the inhibition of myoblast differentiation by MβCD, as demonstrated in this study. It remains to be determined whether these mechanisms act independently or in concert. The first possibility for the inhibition of differentiation in myoblasts by MβCD, as observed in this study, may be attributed to a decrease in the number of dead cells caused by cholesterol depletion (Figure 4). Our experiments on C2C12 myoblast cells grown in a serum-reduced medium revealed that the addition of MβCD significantly reduced the occurrence of apoptotic cell death, which was shown to be dependent on cholesterol (Figures 4A, B). Previous studies have indicated that apoptosis during the induction of differentiation of C2C12 cells leads to cell fusion and subsequent differentiation into myotube (Hochreiter-Hufford et al., 2013). Thus, the inhibition of apoptosis by MβCD may prevent the differentiation of myoblasts into myotube, as depicted in Figures 4, 7. Although cholesterol-dependent apoptosis has been reported in cancer cells and other cells and its mechanism has been partly elucidated (Li et al., 2020), (Lim et al., 2014), (Devries-Seimon et al., 2005), our study is the first to report the role of cholesterol in the apoptosis of C2C12 myoblast cells. Further research is needed to investigate the mechanisms underlying cholesterol-dependent apoptotic cell death in myoblast cells. A second possibility for the inhibition of myoblast differentiation by MβCD could be the disruption of lipid rafts on the plasma membrane due to cholesterol depletion. As lipid rafts are crucial for myoblast differentiation into myotubes, and cholesterol plays a significant role in the formation and maintenance of these rafts (Mukai et al., 2009), (Taulet et al., 2009), it is plausible that MβCD’s action in destroying these rafts could be responsible for blocking the differentiation process. A potential third mechanism for the inhibition of myoblast differentiation by MβCD is the disruption of primary fusion between differentiating myoblasts. Cholesterol in the membrane is critical for the pre-fusion phase triggered by cell fusion (Mukai et al., 2009), (Yang et al., 2016), and cholesterol accumulation has been observed in the plasma membrane of myoblasts or myotubes in C2C12 cells (Figure 3A). Supplementation of MβCD, which depletes cholesterol from the plasma membrane, may therefore hinder membrane fusion between differentiating myoblasts. A fourth potential explanation for the observed phenomenon is that decreased mitochondrial activity may impede the differentiation of myoblasts into myotubes. Through experiments using MβCD supplementation, we have demonstrated that cholesterol depletion leads to the activation of the cholesterol biosynthesis pathway (Figure 3C), as evidenced by increased oxygen consumption. However, this increase in oxygen consumption may also reduce mitochondrial respiration, as the cholesterol biosynthesis pathway competes for oxygen (Kambach et al., 2017). Given that mitochondrial metabolic changes and increased respiration are crucial for myoblast differentiation into myotubes (Remels et al., 2010), (Wagatsuma and Sakuma, 2013), the activation of the cholesterol biosynthesis pathway by MβCD supplementation (Figure 3C) could potentially hinder this process by reducing mitochondrial respiration.

Cell differentiation and proliferation are intricately linked processes (Brown et al., 2003), (Ruijtenberg and van den Heuvel, 2016), with actively growing undifferentiated cells losing their ability to proliferate as differentiation progresses, exiting the cell cycle and entering a quiescent state marked by mitotic arrest (Lehka and Rędowicz, 2020). This phenomenon also occurs during myogenesis in both C2C12 cells (Ruijtenberg and van den Heuvel, 2016) and primary cultured chick embryo muscle cells (Costa et al., 2021), that the inhibitory effects of MβCD supplementation on differentiation from myoblasts to myotubes may also affect the proliferative capacity of myoblasts. Interestingly, MβCD did not appear to have a direct effect on proliferation as a mitogen (Portilho et al., 2012), as enhanced proliferation was only observed in a serum-reduced medium that induces differentiation, but not in a serum-rich medium. These findings suggest that the effect of MβCD on proliferation may be a secondary effect resulting from the inhibition of myoblast differentiation.

The results of this study are expected to have implications for the cost-effective expansion of myoblasts under serum-reduced or -free culture conditions, which are necessary for the mass production of cultured meat in the future. Furthermore, the MβCD used in this study is a cyclic oligosaccharide derived from starch and appears to meet the criteria for food safety. Our observations of cholesterol-dependent induction of apoptotic cell death in myoblasts and the inhibitory effects of MβCD on this process may not only be useful for producing cultured meat, but also for understanding the underlying causes of muscle diseases associated with muscle cell death and developing potential treatments (Amor et al., 2021).

## Data Availability

The datasets presented in this study can be found in online repositories. The names of the repository/repositories and accession number(s) can be found below: DDBJ Search (https://ddbj.nig.ac.jp/search) with accession: DRA014867.

## Supplementary material

SUPPLEMENTARY FIGURE S1 Optimization of MβCD concentration for inhibition of myoblast differentiation on C2C12 cells in DH2 medium conditions. The concentration-dependent inhibitory effect of MβCD on myotube cell differentiation in C2C12 myoblast cells, up to a concentration of 3 mM. However, at concentrations exceeding 3 mM, high cytotoxicity was observed. Despite the occurrence of cytotoxicity, the inhibitory effect on myotube differentiation continued to be observed. The scale bar for the left and center images is 200 µm and for the right images is 66 µm.

SUPPLEMENTARY FIGURE S2 The Gene Ontology Biological Processes (GO_BP) term enrichment analysis. The differentially expressed genes (DEGs) in C2C12 cells under different culture conditions (d0_in DF10, d1_Control, d3_Control, d1_MβCD, d3_MβCD) were analyzed using a hierarchical clustering heatmap with GO terms. This analysis revealed five distinct groups of genes, categorized as cluster 1 to 5 (Figure 2B), and their associated Gene Ontology Biological Process (GO_BP) term enrichment results and annotations were indicated for each cluster.

SUPPLEMENTARY FIGURE S3 Gene expression for lineages other than muscle. The analysis of gene expression for lineage-specific markers in differentiating C2C12 cells was conducted using RNA-sequence data to evaluate the osteogenic and adipogenic differentiation with MβCD treatment. Runx2, Bmpr2, and Sox9 were identified as osteogenic differentiation markers (Sox9, also as a marker for chondrogenic differentiation), while Cebpa, Cebpb, and Pparg were identified as markers for adipogenic differentiation (Robert et al., 2020). The count values were determined by performing TMM normalization on the RNA-sequence data.

SUPPLEMENTARY FIGURE S4 Optimizing cholesterol concentration without cytotoxicity on C2C12 cells in DH2 medium conditions. Above 30 μM, cholesterol precipitations or cytotoxicity started to become noticeable. Furthermore, an inhibitory effect on myotube differentiation was observed at 50 and 100 μM, in addition to the accompanying cytotoxicity. A suitable concentration of 15 µM was determined for further experiments in this report. The scale bar is 200 µm (left and center images) and 66 µm (right images).

SUPPLEMENTARY FIGURE S5 Optimization of MβCD concentration without cytotoxicity on C2C12 cells cultured at low cell density in DH2 medium conditions. At low cell density, 3 mM MβCD caused cytotoxicity, but not at high cell density (Supplementary Figure S1). Additionally, a slight inhibitory effect of MβCD on cell growth was observed at 1 mM. Therefore, the appropriate concentration for further experiments at low cell density in this report was determined to be 0.8 mM. The scale bar in the left and center images is 200 μm, while the right images have a scale bar of 66 µm.

SUPPLEMENTARY FIGURE S6 The effects of MβCD on the proliferation of C2C12 cells cultured in serum-rich conditions. a, The left panel displays representative images of C2C12 cells cultured under DF10 conditions, while the right graph shows the relative cell number of C2C12 cells treated with MβCD on day 3. An inhibitory effect on cell density was observed on days 1 and 3 compared to the control. The scale bar is 200 μm, and the error bars represent s.d. (n = 4, biological replicates). b, FACS analysis for the effect of MβCD on p-Histone H3-positive cells on day 3 cultured in DF10 medium. The proportion of p-Histone H3-positive cells relative to the total cell number as a percentage was calculated with FlowJo. Error bars represent s.d. (n = 3, biological replicates), and the one-way ANOVA followed by the t-test with **p < 0.01.
