# Supplementary figures and images for: The ensured proliferative capacity of myoblast in serum-reduced conditions with Methyl-β-cyclodextrin
Source: Front Cell Dev Biol. 2023 May 12;11:1193634. doi: 10.3389/fcell.2023.1193634 (PMC10213241; doi:10.3389/fcell.2023.1193634)

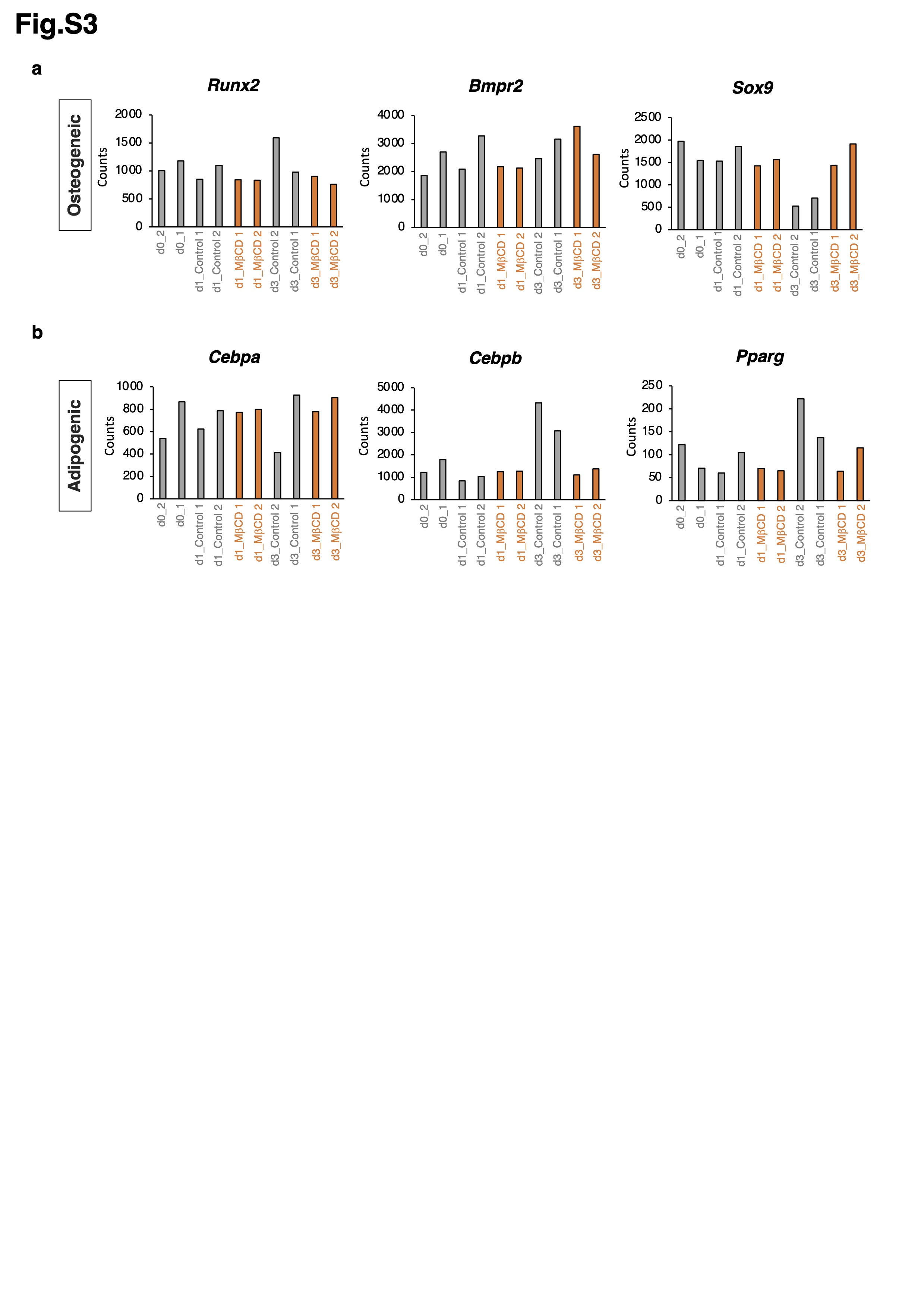

Supplement: Supplementary file 1 [file Image3.JPEG]

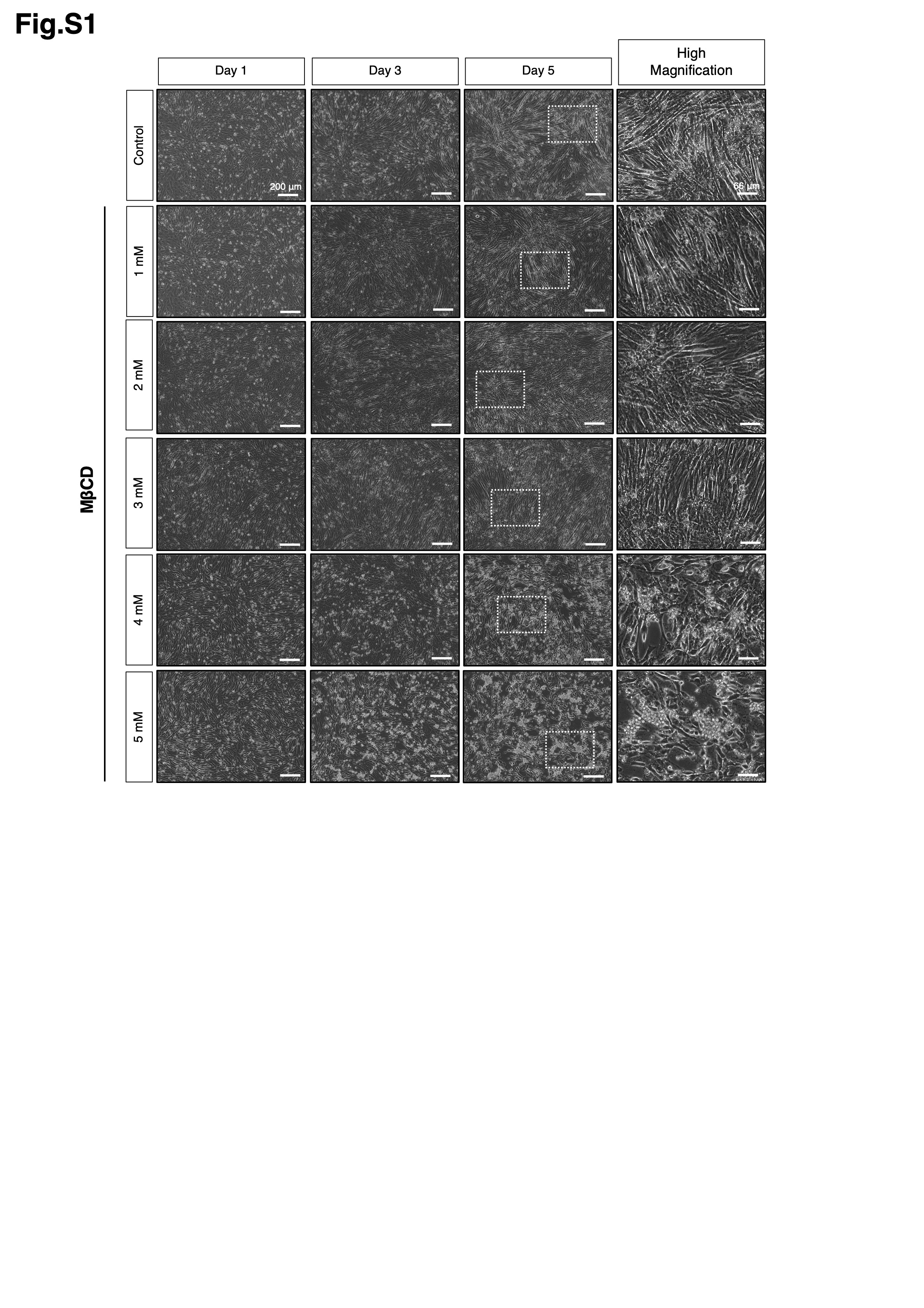

Supplement: Supplementary file 2 [file Image1.JPEG]

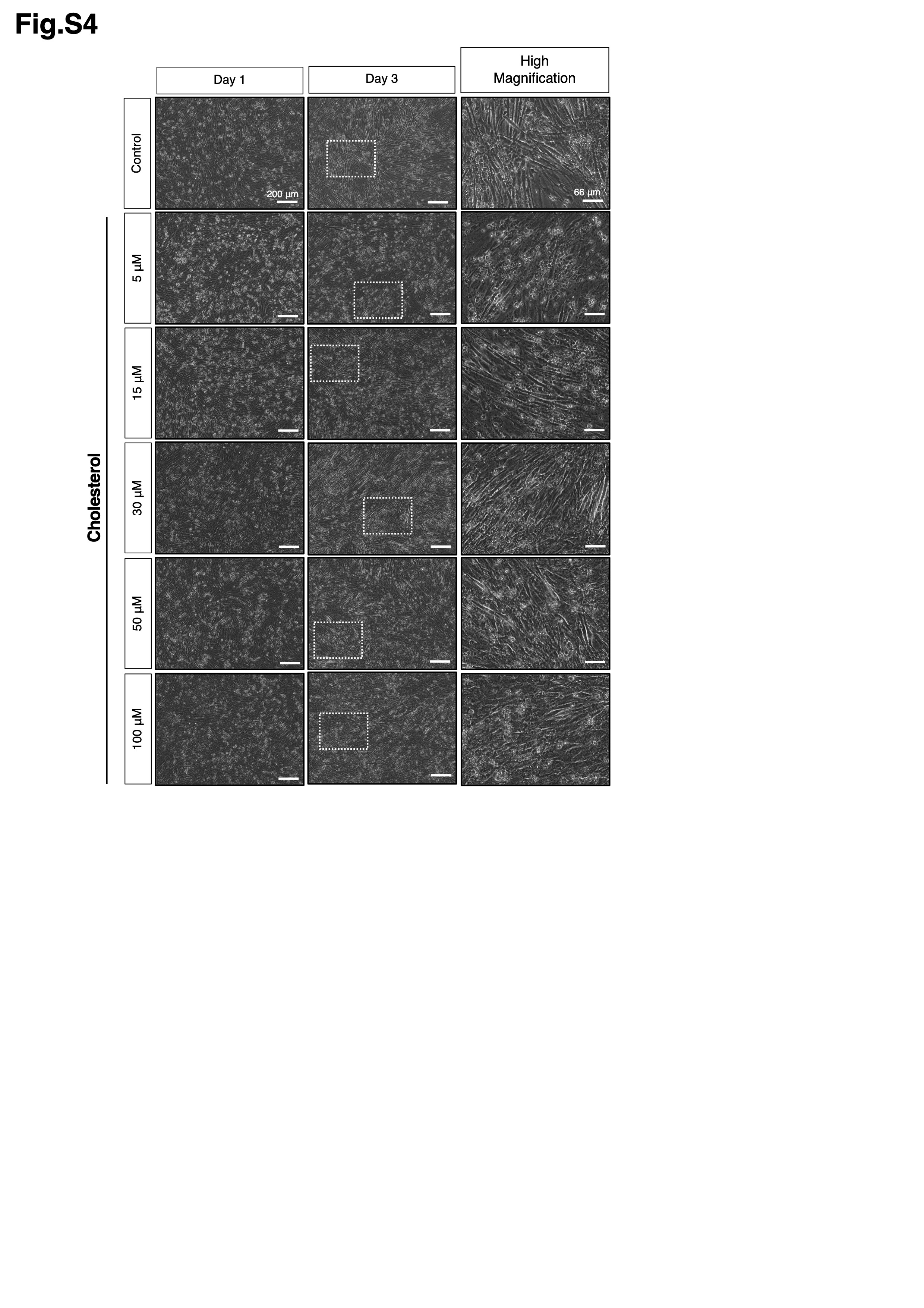

Supplement: Supplementary file 3 [file Image4.JPEG]

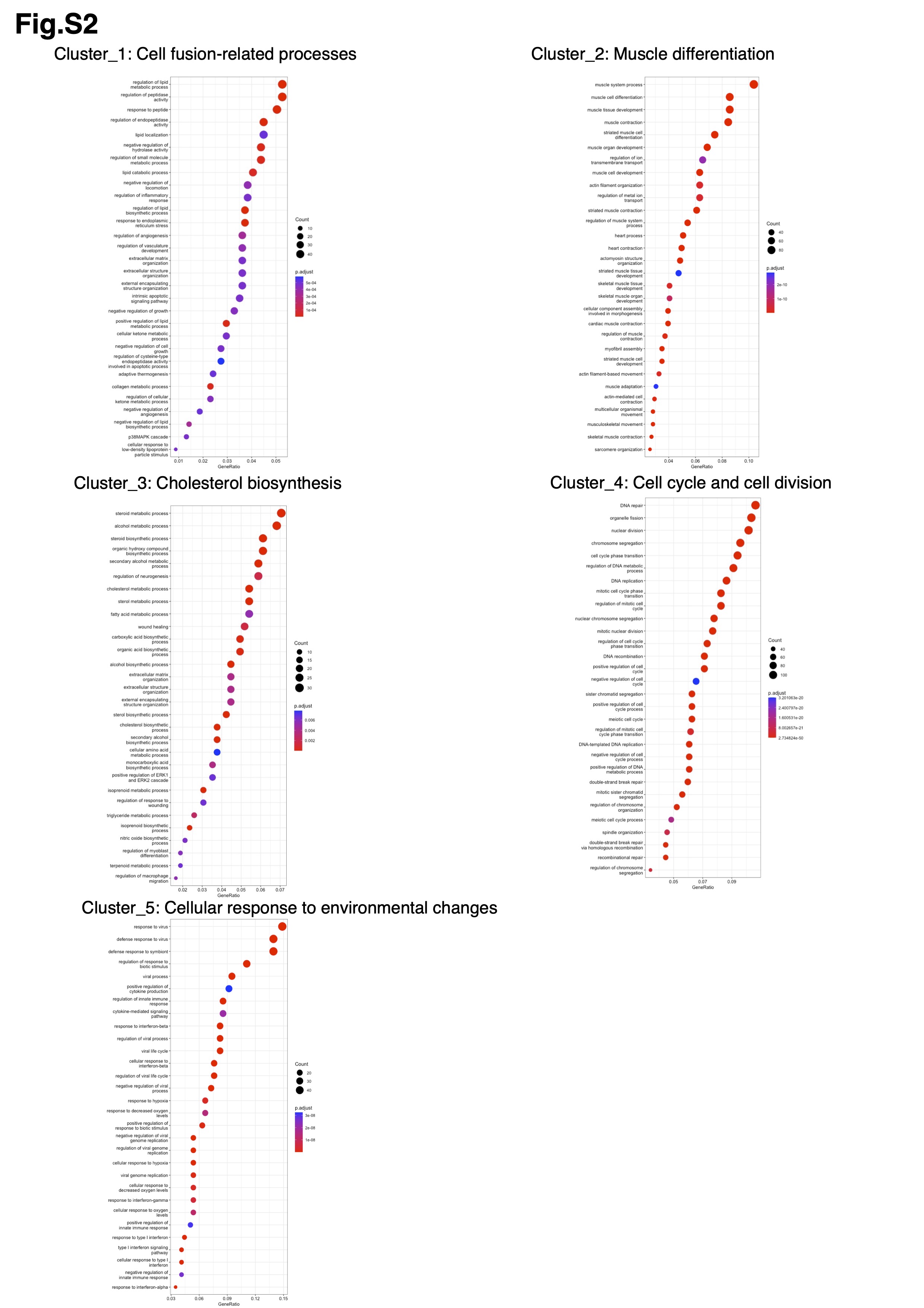

Supplement: Supplementary file 4 [file Image2.JPEG]

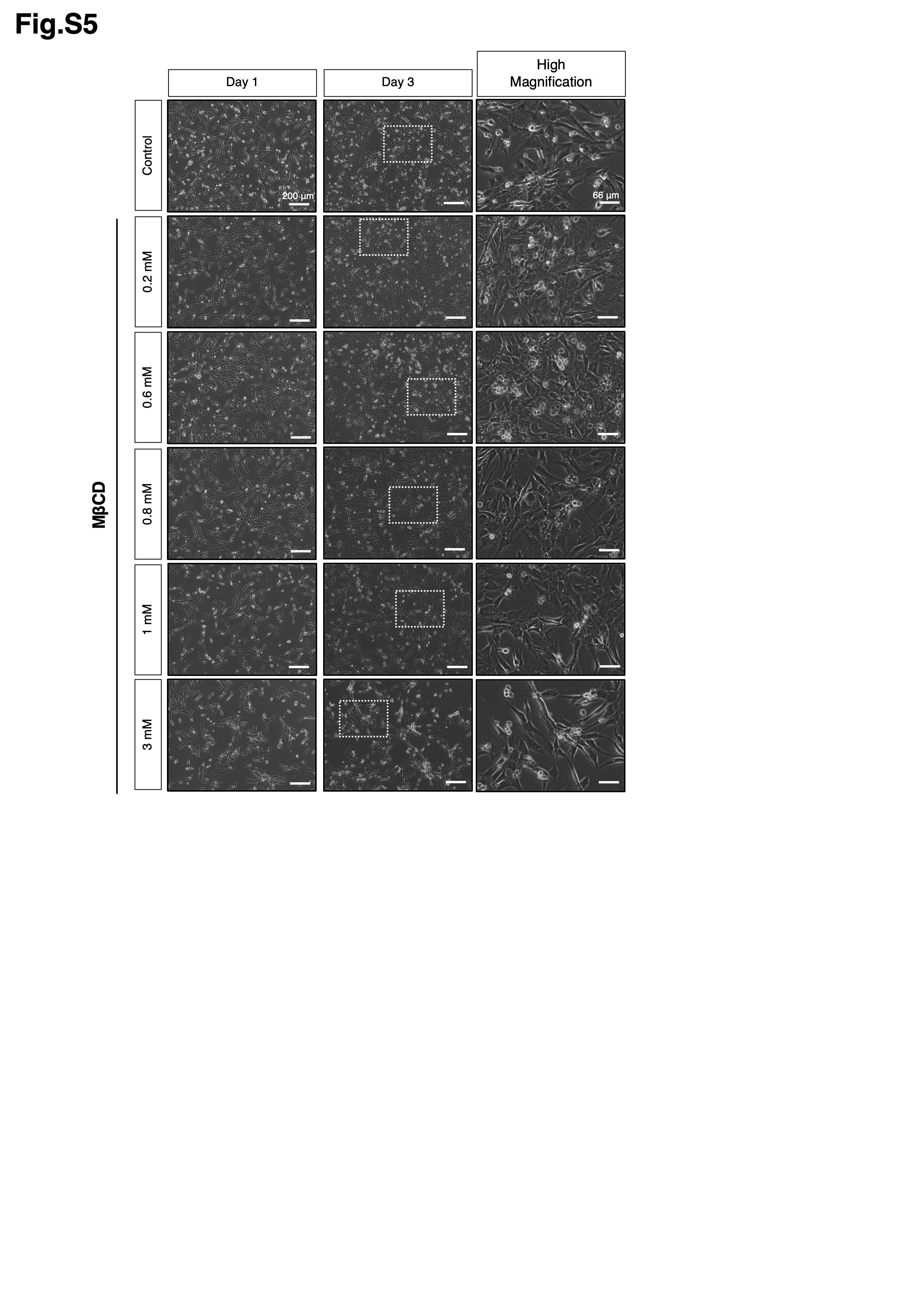

Supplement: Supplementary file 5 [file Image5.JPEG]

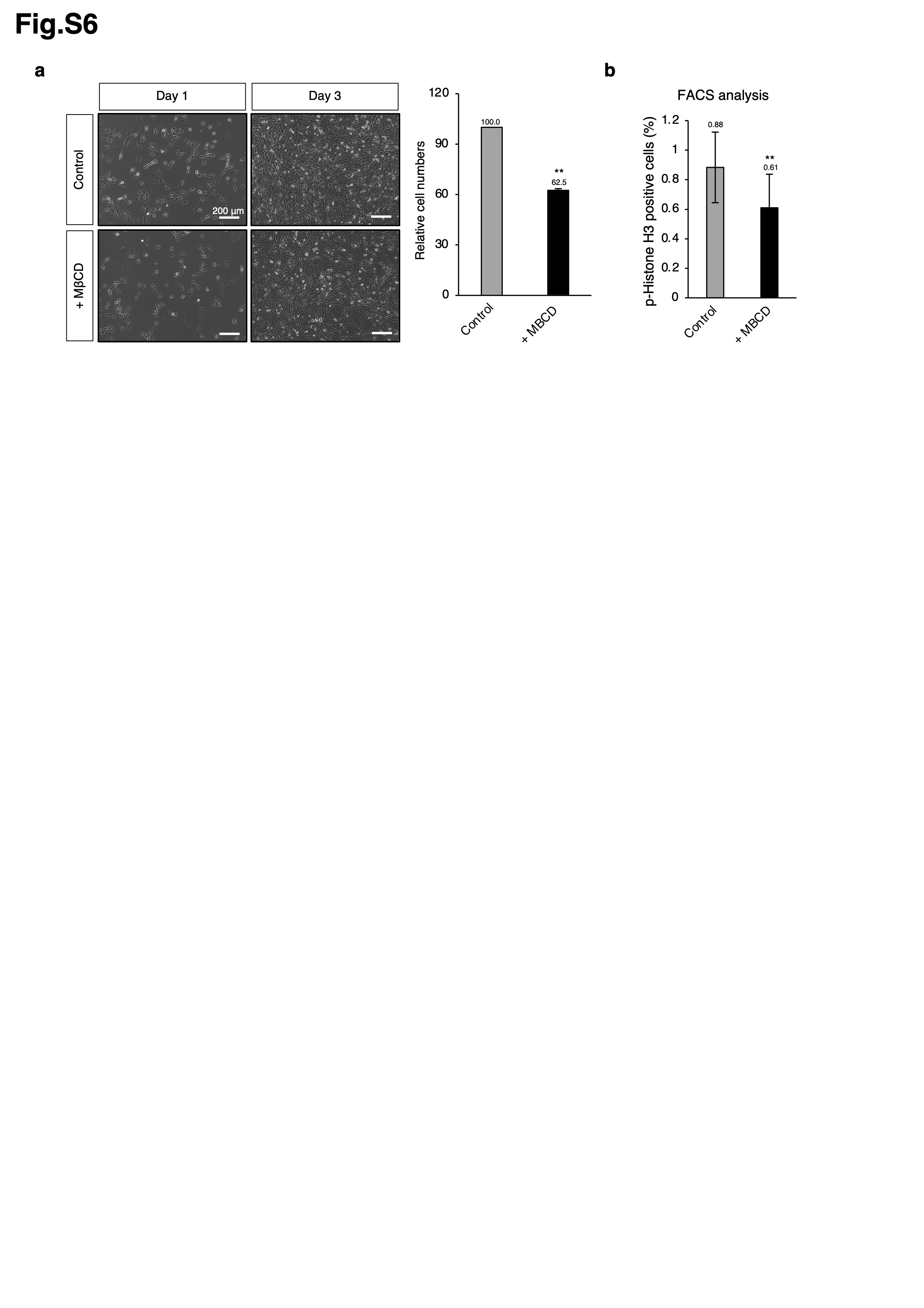

Supplement: Supplementary file 6 [file Image6.JPEG]
